# Supplementary material for: Low-dose carboplatin modifies the tumor microenvironment to augment CAR T cell efficacy in human prostate cancer models
Source: Nat Commun. 2023 Sep 2;14:5346. doi: 10.1038/s41467-023-40852-3 (PMC10475084; doi:10.1038/s41467-023-40852-3)
Supplement: Supplementary file 1 — Supplementary Information [file 41467_2023_40852_MOESM1_ESM.pdf]

Supplementary Figures

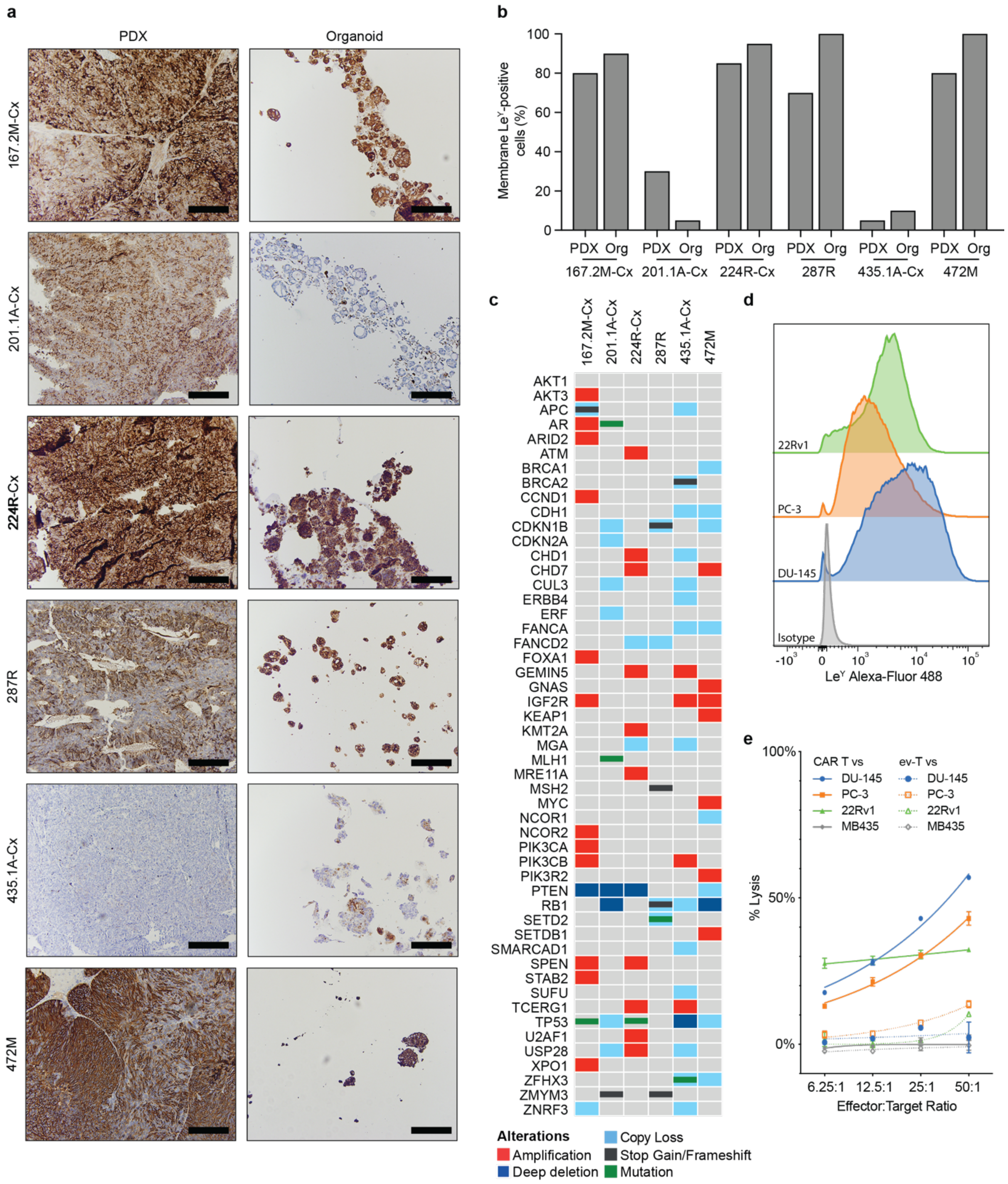

Supplementary Figure 1. On-target killing of Le^{Y}-positive cells in organoids and cell lines in vitro. a Immunohistochemical staining of Le^{Y} in PDXs and organoids (scale bars = 200 µm). PDX-167.2M-Cx, PDX-224R-Cx, PDX-287R and PDX-472M have high membrane staining compared to PDX-201.1A-Cx, which has less

membrane staining, but high cytoplasmic staining. **b** The percentage of cells positive for membrane Le^{Y} expression in PDXs and organoids. **a-b** Staining was repeated on three generations of PDX tissue, and results from the latest generation are shown. **c** Genomic alterations in MURAL PDXs based on targeted DNA sequencing. Somatic nucleotide variations with 0.75 or greater allelic frequency are reported (amplification with 3 or more copies – red; deep deletion – dark blue; copy loss – light blue; stop gains and frameshift mutations – black; missense mutation – green). **d** Representative FACs histograms (representative of n=3 samples/cell line) for the expression of Le^{Y} on prostate cancer cell lines DU-145 (blue), PC-3 (orange), 22Rv1 (green) and isotype control (grey). **e** ^{51}Chromium release assays showing the percentage lysis of Le^{Y+} DU-145 (blue), Le^{Y+} PC-3 (orange), Le^{Y+} 22Rv1 (green) and Le^{Y-} MDA-MB435 (grey) after four hours in co-culture with Le^{Y} CAR T cells (solid lines) or ev-T cells (dot lines) at different effector to target ratios (n=3 wells/cell line). Data represent the mean ± SEM of triplicate cultures. Source data are provided as a Source Data file.

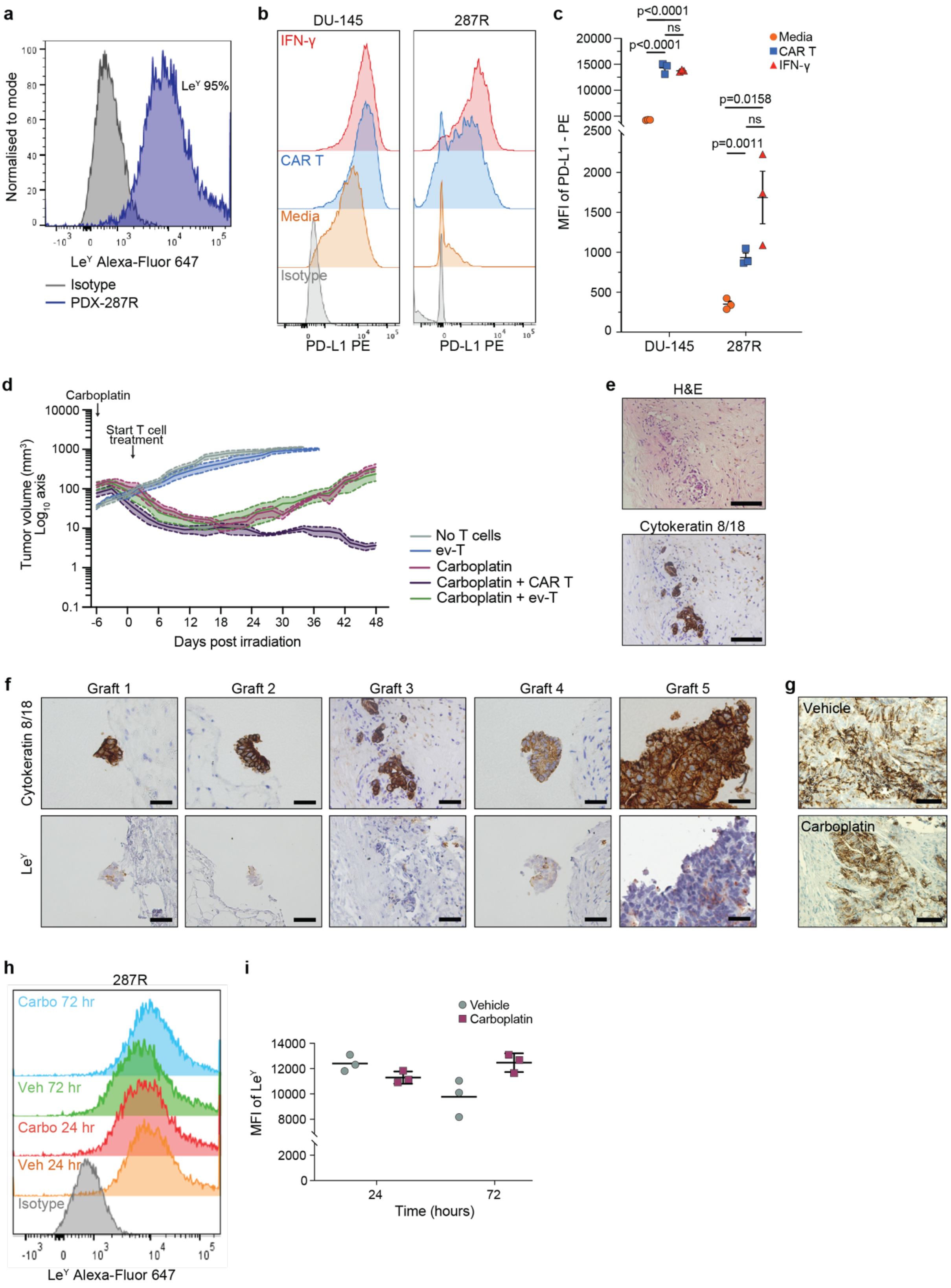
a
Normalised to mode
LeY 95%
LeY Alexa-Fluor 647
Isotype
PDX-287R
b
DU-145
287R
IFN-γ
CAR T
Media
Isotype
PD-L1 PE
c
MFI of PD-L1 - PE
p<0.0001
ns
p<0.0001
p=0.0158
ns
p=0.0011
Media
CAR T
IFN-γ
DU-145
287R
d
Carboplatin
Start T cell treatment
Tumor volume (mm3) Log10 axis
Days post irradiation
No T cells
ev-T
Carboplatin
Carboplatin + CAR T
Carboplatin + ev-T
e
H&E
Cytokeratin 8/18
f
Graft 1
Graft 2
Graft 3
Graft 4
Graft 5
Cytokeratin 8/18
LeY
g
Vehicle
Carboplatin
h
287R
Carbo 72 hr
Veh 72 hr
Carbo 24 hr
Veh 24 hr
Isotype
LeY Alexa-Fluor 647
i
Vehicle
Carboplatin
MFI of LeY
Time (hours)

Supplementary Figure 2. A single dose of carboplatin improves the response to Le^{Y} CAR T cells without altering Le^{Y} expression. a Representative FACs histogram for the expression of Le^{Y} on cells from PDX-287R. Expression of Le^{Y} was 94.5 ± 0.62 % from n = 6 independent samples. **b** Representative FACS histograms (n=3 samples) for the expression of PD-L1 on prostate cancer cell line DU-145 and prostate cancer organoid 287R, treated with CAR T conditioned media (CAR T, in blue), media containing 150 ng/ml IFN-γ (IFN-γ, in red) or fresh media only (Media, in orange). **c** Collective analysis of geometric mean fluorescence intensity (MFI) of PD-L1 on DU-145 cells and 287R organoids (n=3 wells). Data represents the mean ± SEM. Significance was determined by two-tailed unpaired t test. ns, not significant. **d** Tumor volume (mean ± SEM) of PDX-287R grafts following treatment with no T cell control (n=12 grafts), empty vector (ev) T cells (n=6 grafts), carboplatin (50 mg/kg, 1 dose; n=6), carboplatin and CAR T cells (n=5 grafts) and carboplatin and ev-T cells (n=5 grafts). Independent experiment from data presented in Fig. 3d including carboplatin + ev-T cell control group. **e** Representative images of residual tumour cells in PDX grafts (n=5 grafts) following 5-6 weeks of treatment with carboplatin plus Le^{Y} CAR T cells. Hematoxylin and eosin staining and human-specific epithelial cell marker cytokeratin 8/18 indicate large stromal infiltration with limited cancer foci. **f** Immunohistochemical staining of cytokeratin 8/18, showing small cancer foci, and Le^{Y} in matched cancer foci in grafts treated with carboplatin and Le^{Y} CAR T cells (n=5 grafts). **g** Immunohistochemical staining of Le^{Y} in PDX-287R grafts treated with vehicle control (n=3 grafts) or carboplatin (50 mg/kg, single dose; n=3 grafts) three weeks after treatment. **h-i** Representative FACS histograms for the expression of Le^{Y} (**h**) and geometric MFI of Le^{Y} (mean ± SEM), gated to isotype control (**i**), on 287R organoids following treatment with vehicle control or carboplatin (10 μM) for 24 or 72

hours (n = 3 samples/treatment/time point). Scale bars = 50 µm (**e**) and 25 µm (**f-g**).

Source data are provided as a Source Data file.

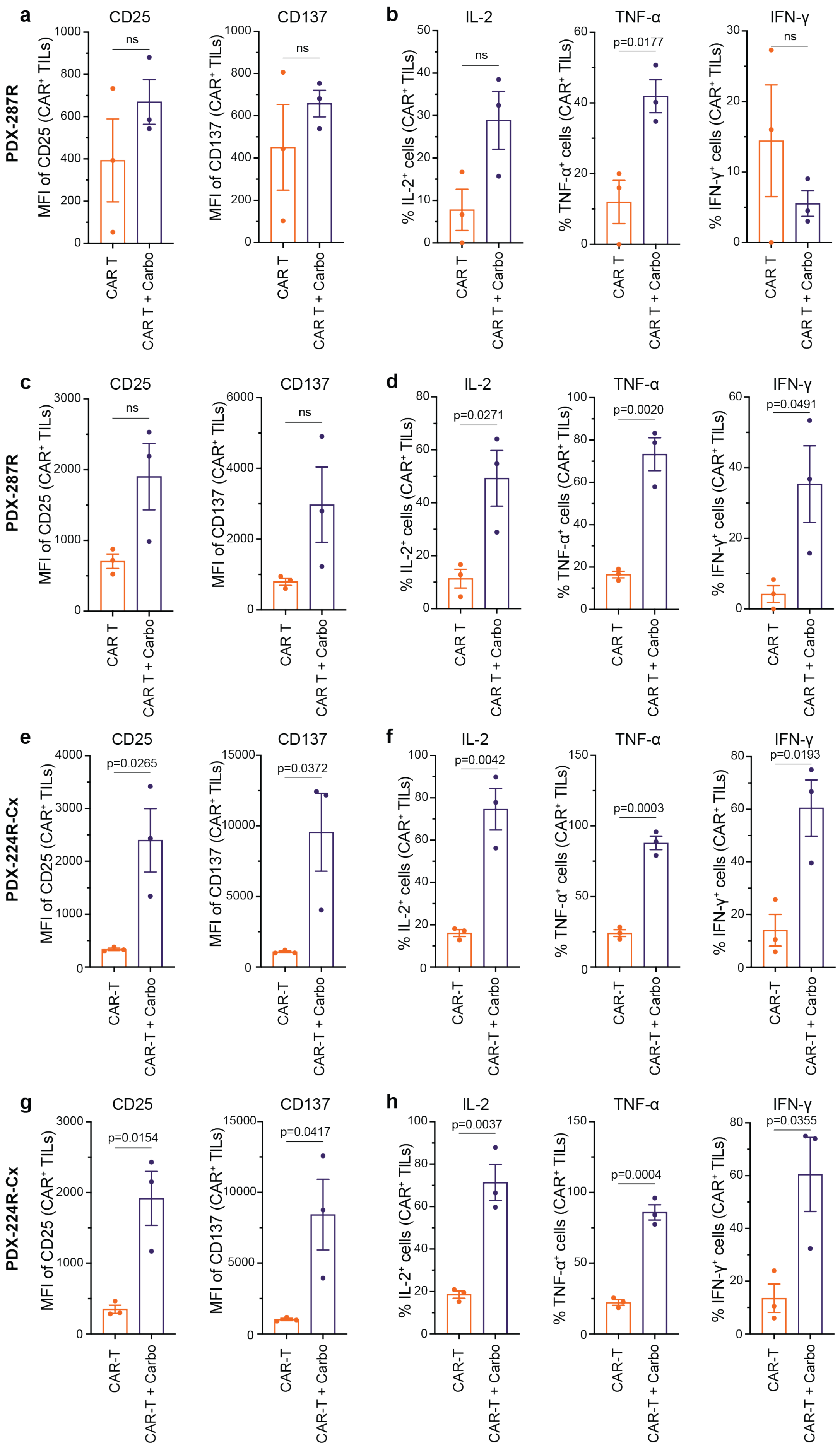
a
b
c
d
e
f
g
h
PDX-287R
PDX-224R-Cx
CD25
CD137
IL-2
TNF-α
IFN-γ
MFI of CD25 (CAR+ TILs)
MFI of CD137 (CAR+ TILs)
% IL-2+ cells (CAR+ TILs)
% TNF-α+ cells (CAR+ TILs)
% IFN-γ+ cells (CAR+ TILs)
ns
p=0.0177
p=0.0271
p=0.0020
p=0.0491
p=0.0265
p=0.0372
p=0.0042
p=0.0003
p=0.0193
p=0.0154
p=0.0417
p=0.0037
p=0.0004
p=0.0355
CAR T
CAR T + Carbo
CAR-T
CAR-T + Carbo

Supplementary Figure 3. Carboplatin increases T cell activation in PDX tumors. a-d Analysis of CAR T cells extracted from tumors (TILs) for PDX-287R without stimulation (**a-b**; cultured with fresh media only) and with TCR stimulation (**c-d**; activation by CD3 mAb clone OKT3) 48 hours post adoptive transfer of CAR T cells by flow cytometry (n = 3 tumors/treatment). **e-h** Analysis of CAR T cells extracted from tumors (TILs) for PDX-224R-Cx without stimulation (**e-f**; cultured with fresh media only) and with TCR stimulation (**g-h**; activation by CD3 mAb clone OKT3) 48 hours post adoptive transfer of CAR T cells by flow cytometry (n = 3 tumors/treatment). Compared to PDX-287R, PDX-224R-Cx had decreased sensitivity to carboplatin treatment alone, and a reduced response to carboplatin-CAR T cell combination treatment. **a,c,e,g** Geometric mean fluorescence intensity (MFI) of CD25 and CD137 without activation (**a,e**; baseline) or with activation by CD3 mAb (**c,g**) on TILs. **b,d,f,h** Percentage of IL-2-, TNF-α-, and IFN-γ-positive TILs without activation (**b,f**; baseline) or with activation by CD3 mAb (**d,h**) using intracellular staining. Data represents the mean ± SEM. Significance was determined by two-tailed unpaired t test. ns, not significant. Source data are provided as a Source Data file.

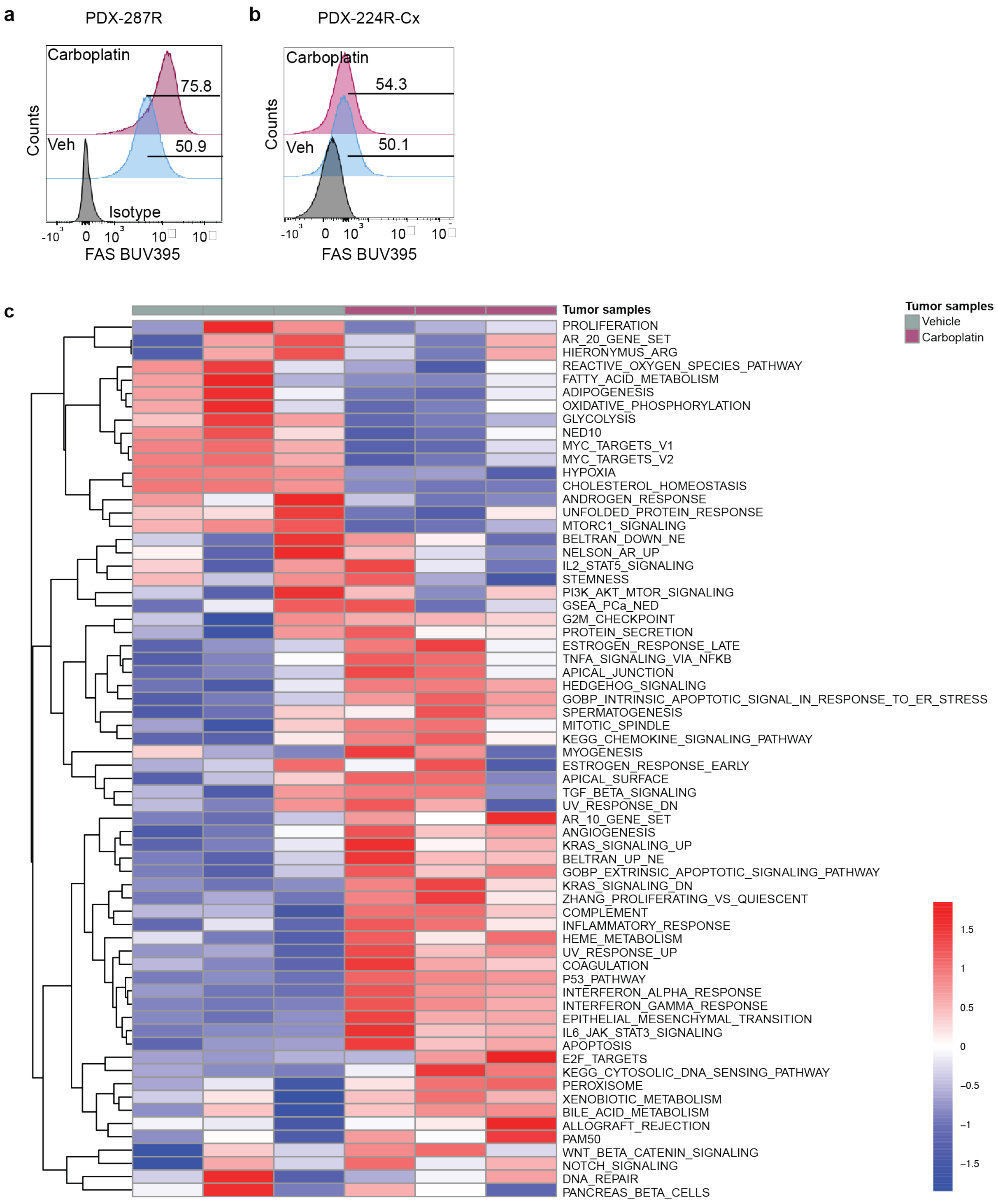
a
PDX-287R
Carboplatin
75.8
Veh
50.9
Isotype
Counts
FAS BUV395
b
PDX-224R-Cx
Carboplatin
54.3
Veh
50.1
Counts
FAS BUV395
c
Tumor samples
Vehicle
Carboplatin
PROLIFERATION
AR_20_GENE_SET
HIERONYMUS_ARG
REACTIVE_OXYGEN_SPECIES_PATHWAY
FATTY_ACID_METABOLISM
ADIPOGENESIS
OXIDATIVE_PHOSPHORYLATION
GLYCOLYSIS
NED10
MYC_TARGETS_V1
MYC_TARGETS_V2
HYPOXIA
CHOLESTEROL_HOMEOSTASIS
ANDROGEN_RESPONSE
UNFOLDED_PROTEIN_RESPONSE
MTORC1_SIGNALING
BELTRAN_DOWN_NE
NELSON_AR_UP
IL2_STAT5_SIGNALING
STEMNESS
PI3K_AKT_MTOR_SIGNALING
GSEA_PCa_NED
G2M_CHECKPOINT
PROTEIN_SECRETION
ESTROGEN_RESPONSE_LATE
TNFA_SIGNALING_VIA_NFKB
APICAL_JUNCTION
HEDGEHOG_SIGNALING
GOBP_INTRINSIC_APOPTOTIC_SIGNAL_IN_RESPONSE_TO_ER_STRESS
SPERMATOGENESIS
MITOTIC_SPINDLE
KEGG_CHEMOKINE_SIGNALING_PATHWAY
MYOGENESIS
ESTROGEN_RESPONSE_EARLY
APICAL_SURFACE
TGF_BETA_SIGNALING
UV_RESPONSE_DN
AR_10_GENE_SET
ANGIOGENESIS
KRAS_SIGNALING_UP
BELTRAN_UP_NE
GOBP_EXTRINSIC_APOPTOTIC_SIGNALING_PATHWAY
KRAS_SIGNALING_DN
ZHANG_PROLIFERATING_VS_QUIESCENT
COMPLEMENT
INFLAMMATORY_RESPONSE
HEME_METABOLISM
UV_RESPONSE_UP
COAGULATION
P53_PATHWAY
INTERFERON_ALPHA_RESPONSE
INTERFERON_GAMMA_RESPONSE
EPITHELIAL_MESENCHYMAL_TRANSITION
IL6_JAK_STAT3_SIGNALING
APOPTOSIS
E2F_TARGETS
KEGG_CYTOSOLIC_DNA_SENSING_PATHWAY
PEROXISOME
XENOBIOTIC_METABOLISM
BILE_ACID_METABOLISM
ALLOGRAFT_REJECTION
PAM50
WNT_BETA_CATENIN_SIGNALING
NOTCH_SIGNALING
DNA_REPAIR
PANCREAS_BETA_CELLS
1.5
1
0.5
0
−0.5
−1
−1.5

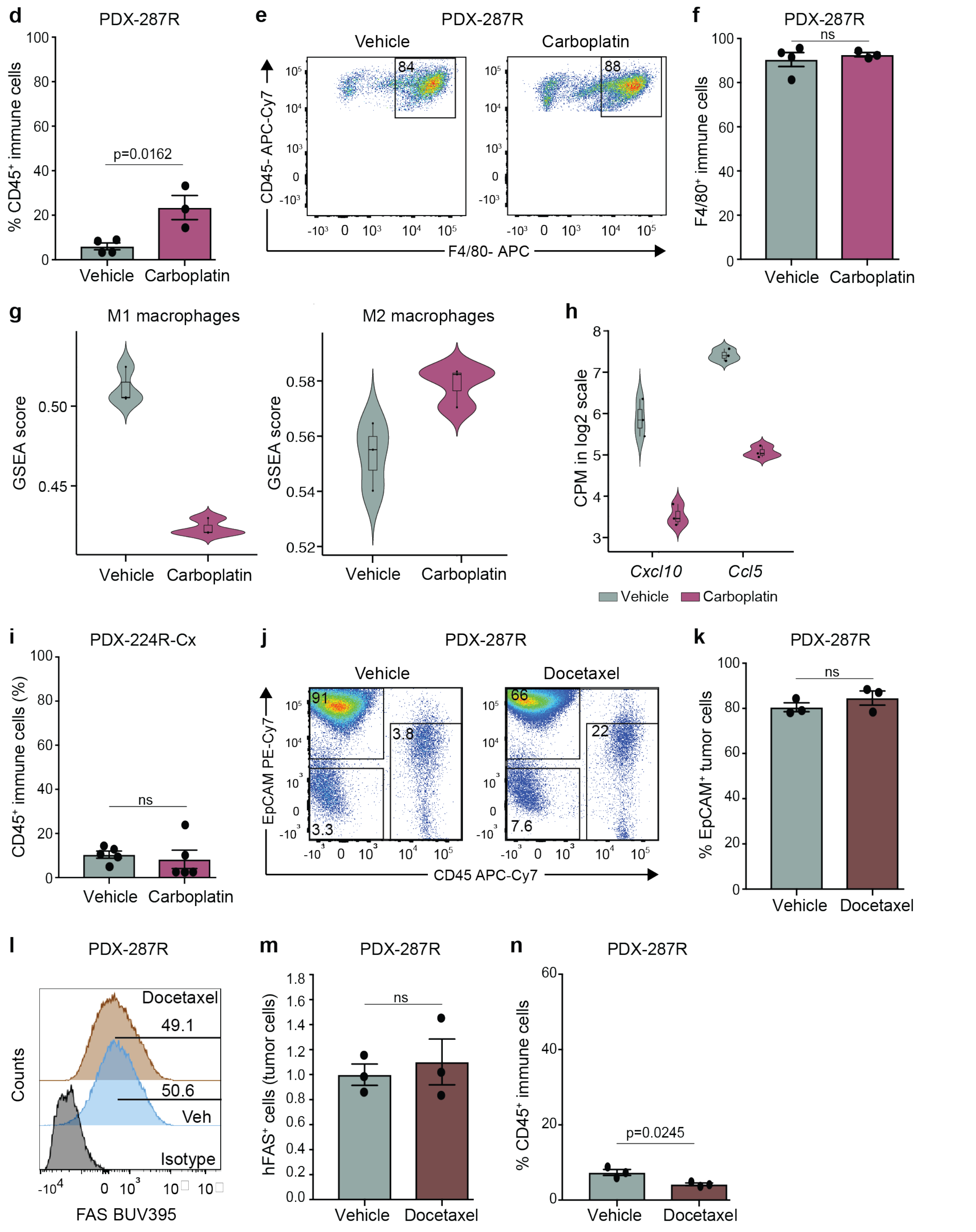

Supplementary Figure 4. Response of PDX-287R and PDX-224R-Cx to carboplatin or docetaxel one week post treatment. a-b Representative FACs histograms for FAS expression on EpCAM⁺ tumour cells in PDX-287R (**a**; n = 4 grafts for vehicle and 3 grafts for carboplatin) and PDX-224R-Cx (**b**; n = 5 grafts/treatment)

one week after treatment with vehicle or carboplatin (50mg/kg, single dose). Compared to PDX-287R, PDX-224R-Cx had decreased sensitivity to carboplatin treatment alone, and a reduced response to carboplatin-CAR T cell combination treatment. **c** Hieratically clustering and heatmap of single-sample gene set enrichment analyses (ssGSEA) based on RNA sequencing for the 50 MSigDB hallmark gene sets plus select KEGG and GOBP gene sets for PDX-287R tumors one week after treatment with vehicle control or carboplatin (n = 3 grafts/treatment). The enrichment of each gene set is shown. **d** The proportion of $CD45^+$ immune cells in PDX-287R grafts after one week treatment with vehicle (n=4 grafts) or carboplatin (n=3 grafts) by flow cytometry. **e** Representative flow cytometry plot showing F4/80 expression in CD45+ immune cells in PDX-287R grafts one week after treatment with vehicle (n=4 grafts) or carboplatin (n=3 grafts). **f** The proportion of $F4/80^+$ immune cells of the myeloid monocytic lineage out of $CD45^+$ immune cells in PDX-287R grafts one week after treatment with vehicle (n=4 grafts) or carboplatin (n=3 grafts). **g-h** Violin plots showing GSEA score enriched proportions of M1 and M2 macrophages (**g**) and the expression of *Ccl5* and *Cxcl10* in immune cells (**h**) within carboplatin-treated and vehicle PDX-287R grafts (n=3 grafts/treatment). GSEA statistical and empirical evaluation were used in **g** to yield a normalized enrichment score (NES; significance level of 5%), and significance was determined by Welch's T-test. Box plots in **f-g** show the first to third quartile with median, whiskers show the minimum and maximum. **i** The proportion of $CD45^+$ immune cells in PDX-224R-Cx grafts after one week treatment with vehicle or carboplatin by flow cytometry (n = 5 grafts/treatment). **j** Representative flow cytometry plot (n = 3 grafts/treatment) showing proportions of $EpCAM^+$ epithelial tumor cells, $CD45^+$ immune cells and $EpCAM^-CD45^-$ stromal cells in PDX-287R grafts one week after treatment with vehicle or docetaxel (10 mg/kg, single dose). **k** The

proportion of $EpCAM^+$ epithelial tumor cells in PDX-287R grafts after one week treatment with vehicle or docetaxel (n = 3 grafts/treatment). **l** Representative FAC histograms for FAS expression on $EpCAM^+$ tumour cells in PDX-287R grafts one week after treatment with vehicle or docetaxel (n=3 grafts/treatment). **m-n** $hFAS^+$ tumor cells (**m**; normalized to average vehicle) and $CD45^+$ immune cells (**n**) in PDX-287R grafts one week after treatment with vehicle or docetaxel (n=3 grafts/treatment). Data in **d,f,i,k,m,n** represents the mean ± SEM, and the significance was determined by two-tailed unpaired t test. ns, not significant. Source data are provided as a Source Data file.

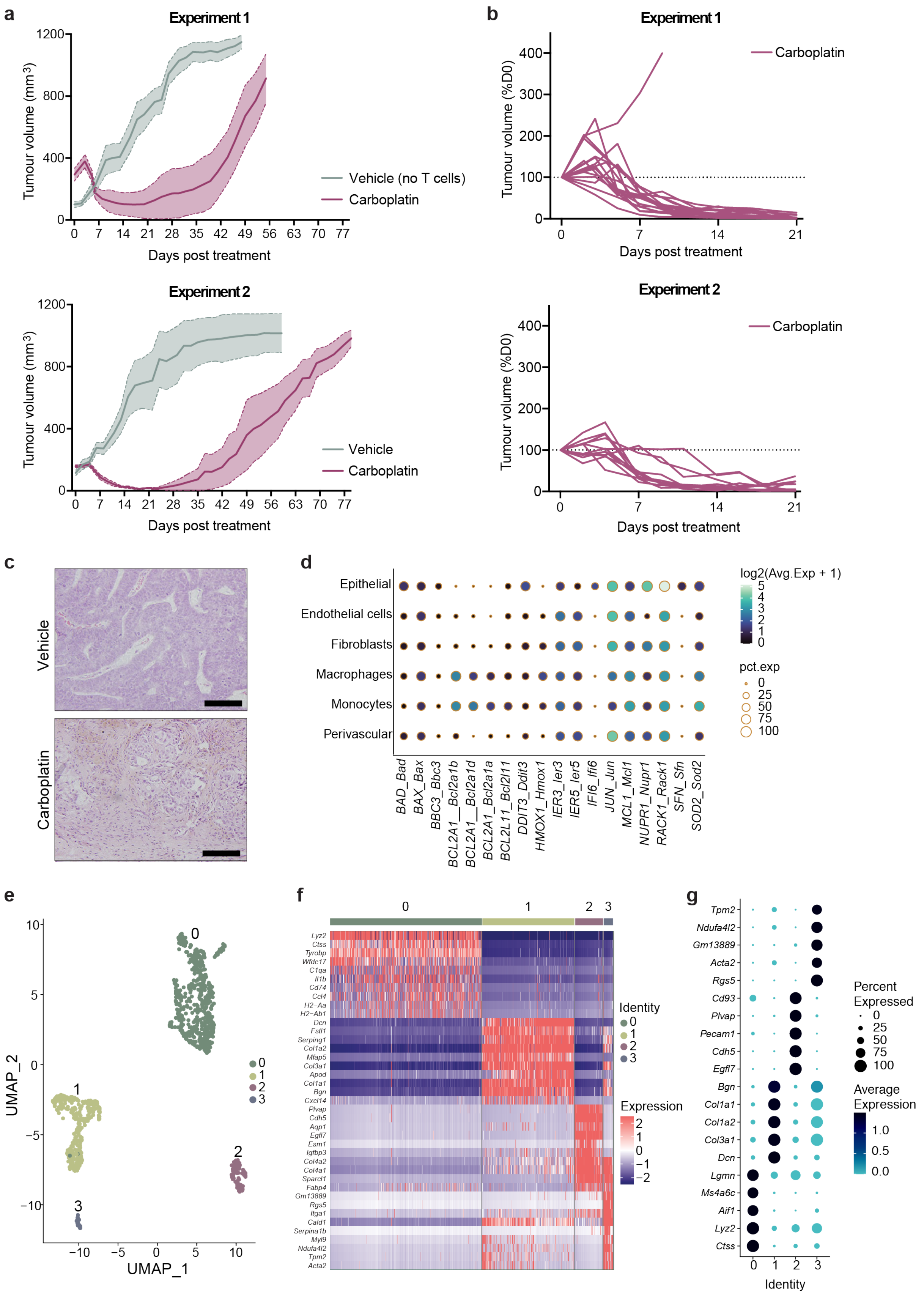

Supplementary Figure 5. Single cell RNA sequencing of PDX-287R three weeks post carboplatin treatment demonstrates a shift to a pro-inflammatory

phenotype. a Tumour volume (mean ± SEM) of PDX-287R grafts following treatment with vehicle or carboplatin (single 50 mg/kg dose at day 0). Experiment 1 is data from Fig. 3d (n=15 grafts for vehicle, n=17 grafts for carboplatin; data from 2 independent experiments). Experiment 2 is tumor changes across time for treatment groups in Fig. 5a (n = 7 grafts for vehicle, n=12 grafts for carboplatin; data from one independent experiment). A subset of grafts from vehicle and carboplatin treatment groups in experiment 2 were harvested three weeks post treatment for ssRNA-seq. **b** Percent change in tumour volume from day 0 of carboplatin treatment (%D0) for individual grafts in experiment 1 (n = 17 grafts) and experiment 2 (n = 12 grafts) for three weeks following treatment. **c** Representative histological hematoxylin and eosin staining of PDX tissue one week post-carboplatin treatment (n=3 grafts), compared to vehicle control (n=2 grafts). Scale bar = 100 µm*.* **d** Relative gene expression of pro- and anti-apoptotic agents by human epithelial cells and murine stroma following carboplatin-treatment when compared to control tissue. **e** UMAP clustering of cellular populations isolated from PDX tissue (0-4). **f** Heatmap illustrating the top 10 differentially expressed (DE) genes defining cellular clusters. **g** Primary genetic markers utilised to define cell type populations utilising a ROC test. Source data are provided as a Source Data file.

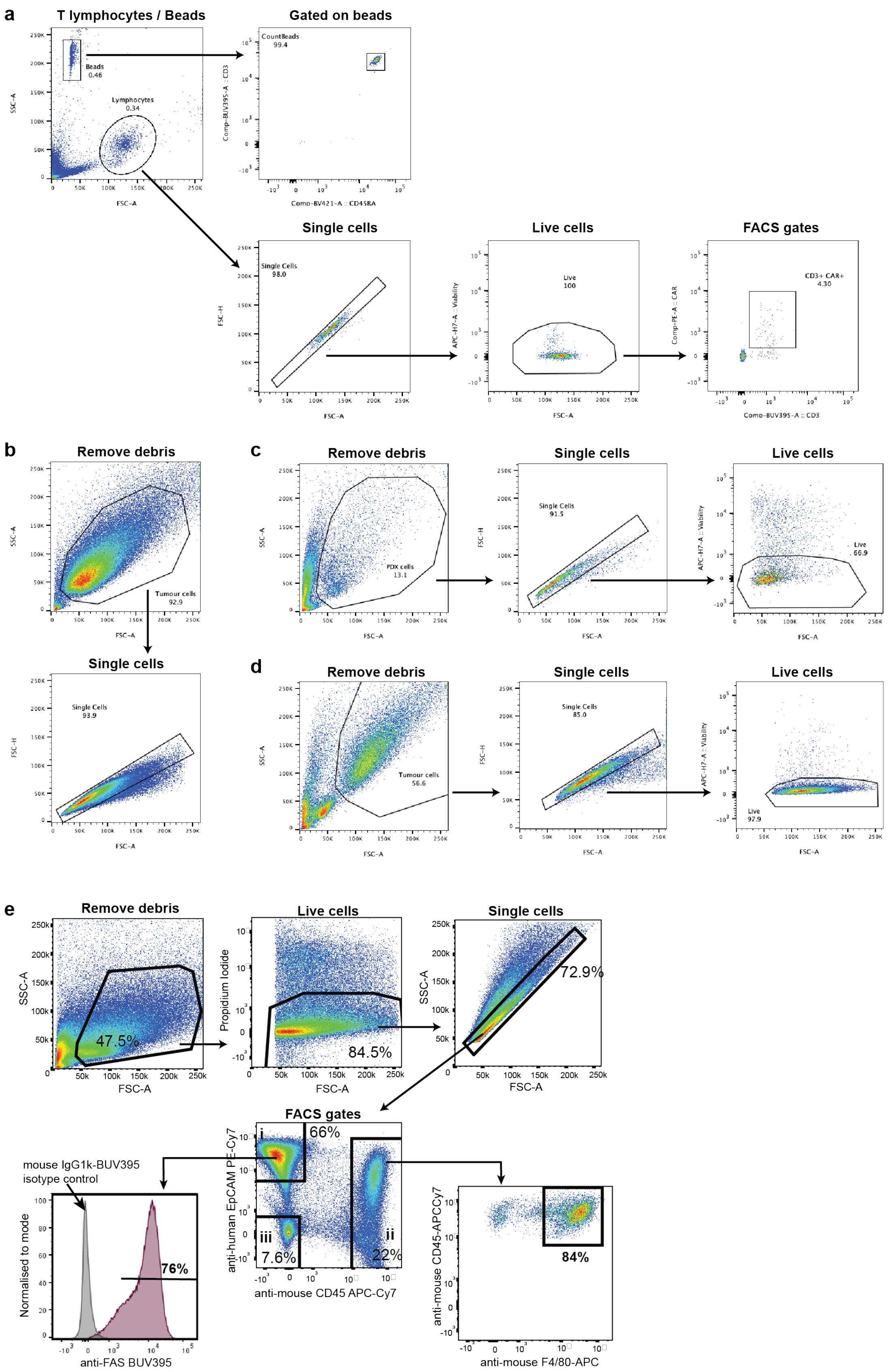

Supplementary Figure 6. Gating strategies for flow cytometry analysis and cell sorting. a Gating strategy for **Fig. 4g-l** and **Supplementary Fig. 3a-h**. Counting

beads were gated by size and positive fluorescence activity for BUV395 and BV421. CAR-T cells were gated by size, single cells, viability and positive fluorescence activity for CD3-BUV395 and CAR-PE. **b** Gating strategy for **Supplementary Fig. 1d**. Tumor cells were pre-tested for 100% viability by Trypan Blue staining (0.4%). Tumor cells were gated by size and single cells. **c** Gating strategy for **Supplementary Fig. 2a,h-l** and PDX-287R cells in **Supplementary Fig. 2b-c**. PDX tumors were digested and resuspended in a single cell suspension. PDX tumor cells were gated by size, single cells and viability. **d** Gating strategy for DU-145 tumor cells in **Supplementary Fig. 2b-c**. Tumor cells were gated by size, single cells and viability. **e** Gating strategy for **Fig. 5a-f** and **Supplementary Fig. 4a,b,d-f,i-n**. Cells were gated on SSC/FSC to exclude debris. Cells were then gated based on exclusion of propidium iodine to gate live cells. Single cells were then gated based on FSC-H and FSC-A properties. Final sort fates for i. Human prostate tumor cells; ii. Murine immune cells, and; iii. Murine stromal cells. EpCAM+ human tumor cells were sorted for FAS and CD45+ murine immune cells were sorted for F4/80.
