# Supplementary material for: Low-dose carboplatin modifies the tumor microenvironment to augment CAR T cell efficacy in human prostate cancer models
Source: Nat Commun. 2023 Sep 2;14:5346. doi: 10.1038/s41467-023-40852-3 (PMC10475084; doi:10.1038/s41467-023-40852-3)
Supplement: Supplementary file 3 — Reporting Summary [file 41467_2023_40852_MOESM3_ESM.pdf]

nature portfolio

Corresponding author(s): Gail P. Risbridger
Renea A. Taylor

Last updated by author(s): 19/6/23

Reporting Summary

Nature Portfolio wishes to improve the reproducibility of the work that we publish. This form provides structure for consistency and transparency in reporting. For further information on Nature Portfolio policies, see our Editorial Policies and the Editorial Policy Checklist.

Statistics

For all statistical analyses, confirm that the following items are present in the figure legend, table legend, main text, or Methods section.

n/a	Confirmed	
☐	☒	The exact sample size (*n*) for each experimental group/condition, given as a discrete number and unit of measurement
☐	☒	A statement on whether measurements were taken from distinct samples or whether the same sample was measured repeatedly
☐	☒	The statistical test(s) used AND whether they are one- or two-sided<br>*Only common tests should be described solely by name; describe more complex techniques in the Methods section.*
☒	☐	A description of all covariates tested
☐	☒	A description of any assumptions or corrections, such as tests of normality and adjustment for multiple comparisons
☐	☒	A full description of the statistical parameters including central tendency (e.g. means) or other basic estimates (e.g. regression coefficient) AND variation (e.g. standard deviation) or associated estimates of uncertainty (e.g. confidence intervals)
☐	☒	For null hypothesis testing, the test statistic (e.g. *F*, *t*, *r*) with confidence intervals, effect sizes, degrees of freedom and *P* value noted<br>*Give P values as exact values whenever suitable.*
☒	☐	For Bayesian analysis, information on the choice of priors and Markov chain Monte Carlo settings
☒	☐	For hierarchical and complex designs, identification of the appropriate level for tests and full reporting of outcomes
☒	☐	Estimates of effect sizes (e.g. Cohen's *d*, Pearson's *r*), indicating how they were calculated

Our web collection on statistics for biologists contains articles on many of the points above.

Software and code

Policy information about availability of computer code

Data collection	No software was utilized for data collection within the manuscript.
Data analysis	Data analysis tools utilized within the manuscript include the following:<br>1. In vitro CAR T killing assay images were analyzed using Fiji (ImageJ; v2.9.0/1.53t) and Bio-formats (v6.10.1) software.<br>2. Flow cytometry data was then analyzed using FlowJo v10.8.1 software.<br>3. Quantification of positive IHC-staining was facilitated through the use of Aperio ImageScope Software (Leica Biosystems, v12.3)<br>4. The raw Fastq files for multiplex RNA-seq were quality checked using FastQC (v0.11.9)<br>5. Low-quality multiplex RNA-seq reads were trimmed using Cutadapt v1.7.1<br>6. Trimmed reads were aligned to both human hg38 and mouse mm39 reference genomes using STAR aligner v2.7.5b or GRCh38 human reference genome and mm10 mouse genome using XenoCell v1.0.<br>7. XenofilteR v1.6 was used to select Human and mouse specific reads.<br>8. Counts matrix were generated using HTSeq v0.11.2.<br>9. EdgeR v3.28 was used for differential expression analysis.<br>10. The normalized log transformed counts per million (CPM) was used to calculate single-sample gene set enrichment analysis score against specific MsigDB signature gene sets using R v4.2.0 package.<br>11. Extracted mouse cells were then processed using Alevin tool (Salmon Software v1.3.0) to obtain unique molecular identifiers and generate a cell by gene count matrix.<br>12. Downstream single-cell RNA-sequencing analysis was performed on Seurat (v3.2.0).<br>13. The ClusterTree (v0.4.3) R package was used to determine the optimal resolution and number of clusters for each sample.<br>14. Gene set enrichment analysis was performed using escape (v1.2.0) enrichIT function in R (v4.1.0).

15. Linear mixed model analyses were conducted using SPSS Statistics (v27).
16. All other statistical analyses were performed using GraphPad Prism v9 software.

For manuscripts utilizing custom algorithms or software that are central to the research but not yet described in published literature, software must be made available to editors and reviewers. We strongly encourage code deposition in a community repository (e.g. GitHub). See the Nature Portfolio guidelines for submitting code & software for further information.

Data

Policy information about availability of data

All manuscripts must include a data availability statement. This statement should provide the following information, where applicable:

- Accession codes, unique identifiers, or web links for publicly available datasets
- A description of any restrictions on data availability
- For clinical datasets or third party data, please ensure that the statement adheres to our policy

All source data are provided in the manuscript.

Data availability statement:
The targeted DNA sequencing, bulk RNA-sequencing, and single-cell RNA-sequencing data that support the findings of this study have been deposited in the NCBI's dbGaP (https://www.ncbi.nlm.nih.gov/gap/). The curated set of genomic alterations was based on data downloaded from the cBioPortal resource Prostate Adenocarcinoma24,25 [https://www.cbioportal.org/ study/summary?id=prad_p1000 and https://www.cbioportal.org/study/summary? id=prad_su2c_2015]. Source data are available as Source Data File. The remaining data are available within the Article, Supplementary Information or available from the reviewers upon request. To request access to MURAL PDXs and/or biospecimens, researchers should contact Dr. Melissa Papargiris, MURAL Project Manager (melissa.papargiris@monash.edu) to initiate an Expression of Interest. Researchers would need to provide evidence of institutional approval to experiment with human PDX tumors and research would be conducted under the conditions of a Materials Transfer Agreement.

Human research participants

Policy information about studies involving human research participants and Sex and Gender in Research.

Reporting on sex and gender	Patients with prostate cancer are of male sex and therefore no patients of female sex were included.
Population characteristics	N/A as this study used patient-derived xenografts (PDXs) from patient specimens that were obtained by informed consent.
Recruitment	All human tissues were obtained with informed, written consent by an independent clinical coordinator.
Ethics oversight	Patient tissue collection was conducted according to human ethics approval from the Cabrini Institute (03-14-04-08), Monash University (1636) and Peter MacCallum Cancer Centre (15/98, 97_27).

Note that full information on the approval of the study protocol must also be provided in the manuscript.

Field-specific reporting

Please select the one below that is the best fit for your research. If you are not sure, read the appropriate sections before making your selection.

[x] Life sciences [ ] Behavioural & social sciences [ ] Ecological, evolutionary & environmental sciences

For a reference copy of the document with all sections, see nature.com/documents/nr-reporting-summary-flat.pdf

Life sciences study design

All studies must disclose on these points even when the disclosure is negative.

Sample size	No optimal sample size calculations were performed. Cohort sample sizes (n) were determined based on the requirements to sufficient power experiments (organoids, single cell RNA-sequencing and flow cytometry) and in accordance with the ethical consideration of minimal animal usage. With n = 5 mice/group, there is 85% power to detect a 1.8-fold difference (α=0.05/3; CV=35%; 1-sided t test). This was the minimum number of mice used in any experimental group (n=5 Fig xx; n=17 Fig xx).
Data exclusions	No patients or animals were excluded. Cells were excluded according to the following criteria; expressed in the range of <200-800 genes depending on the sample type and had an unusual gene count, transcript count and mitochondrial gene fraction, according to sample-specific thresholds (Supplementary Table 1). Genes were excluded on the basis that their expression was limited to fewer than 50 cells. Vehicle-control treated epithelial tumor cells were down-sampled through randomized selection to 100 cells, similar to the amount of the carboplatin sample (99 cells).
Replication	In vitro organoid experimentation was conducted in triplicates, and data were reproducible between wells.
Randomization	For in vivo PDX treatments, mice were randomly allocated to treatments groups as grafts reached approximately 100mm3 in volume.

Blinding	The investigators involved in treating mice were not blinded to the treatment groups but were blinded to tumor measurement data. Tissues for histology were blinded during staining and subsequent analysis. Single cell RNA seq and RNA seq data were analysed according to sample type. Treatments for organoid cultures were not blinded, but the correlation to LeY expression was blinded.

Reporting for specific materials, systems and methods

We require information from authors about some types of materials, experimental systems and methods used in many studies. Here, indicate whether each material, system or method listed is relevant to your study. If you are not sure if a list item applies to your research, read the appropriate section before selecting a response.

Materials & experimental systems

n/a	Involved in the study	
☐	☒	Antibodies
☐	☒	Eukaryotic cell lines
☒	☐	Palaeontology and archaeology
☐	☒	Animals and other organisms
☒	☐	Clinical data
☒	☐	Dual use research of concern

Methods

n/a	Involved in the study	
☒	☐	ChIP-seq
☐	☒	Flow cytometry
☒	☐	MRI-based neuroimaging

Antibodies

Antibodies used

1. Primary Lewis Y antibody (m3S193; optimization protocol cited in PMID: 19242371)
2. Primary phospho-histone H3 antibody (Ser10; Cell Signaling Technology, #9701, polyclonal, lot #17).
3. Primary CD3 antibody (Abcam, #ab16669, clone SP7, lot #GR3253342-1 for IHC; BD Biosciences, #564001, clone SK7 for flow cytometry).
4. CD8 antibody (Invitrogen, #MA1-80231, clone 4B11, lot #VC295499 for IHC; BD Biosciences, #612889, clone SK1 for flow cytometry).
5. Primary F4/80 antibody (Abcam, #100790, polyclonal, lot #GR282642-1)
6. Secondary anti-mouse ImmPress (Vector Laboratories, MP-7402)
7. Secondary anti-rabbit ImmPress (Vector Laboratories, MP-7401)
8. Primary CD4 antibody (BioLegend, #317438, clone OKT4)
9. PE anti-DYKDDDDK Tag Antibody for CAR identification (BioLegend, #637310, clone L5)
10. Primary CD137 antibody (BioLegend, #309814, clone 4B4-1)
11. Primary CD25 antibody (BioLegend, #302629, clone BC96)
12. IgG3-APC isotype control (optimization protocol cited in PMID: 1768791)
13. Rat anti-mouse CD16/CD32 (BD Bioscience, #553141, clone 2.4G2, lot #7248907)
14. Anti-human EpCAM-PECy7 antibody (BioLegend, #324221, clone 9C4, lot #B371004)
15. Anti-human CD95-BUV395 (BD Biosciences, #740306, clone DX2, lot #2159594)
16. Anti-mouse MHC I-Ak-PE (BioLegend, #109908, clone 10-3.6, lot #B363881)
17. Anti-mouse CD45-APCCy7 (BioLegend, #103115, clone 30-F11, lot #B370047)
18. Anti-mouse ICAM1-BV421 (BioLegend, #116141, clone YN1/1.7.4, lot #B359248)
19. Anti-mouse CD31-BV510 (BD Biosciences, #740124, clone 390, lot #3031793)
20. Mouse IgG1k-BUV395 (BD Biosciences, #563547, clone X40, lot #1172135)
21. Rat IgG2bk-BV421 (BioLegend, #400655, clone RTK4530)
22. Anti-mouse F4/80-APC (BioLegend, #123116, clone BM8)

Validation

See antibodies validated through commercial production below:
2. Primary phospho-histone H3 antibody validated for IHC (paraffin) by CST according to protocol ID: 283 [https://www.cellsignal.com/products/primary-antibodies/phospho-histone-h3-ser10-antibody/9701]
3. Primary CD3 antibody application for IHC-P has been assessed by Abcam and further dilution has been optimized internally, as recommended [https://www.abcam.com/products/primary-antibodies/cd3-antibody-sp7-ab16669.html]
4. CD8 antibody application for IHC-P has been assessed by Thermo Fisher Scientific [https://www.thermofisher.com/antibody/product/CD8-Antibody-clone-4B11-Monoclonal/MA1-80231]
5. Primary F4/80 antibody application for IHC-P has been assessed by Abcam and further dilution has been optimized internally, as recommended [https://www.abcam.com/products/primary-antibodies/f480-antibody-ab100790.html]
6. Secondary anti-mouse ImmPress application for IHC has been assessed by Vector Laboratories [https://vectorlabs.com/products/enzyme-polymer/immpress-hrp-horse-anti-mouse-igg-kit]
7. Secondary anti-rabbit ImmPress application for IHC has been assessed by Vector Laboratories [https://vectorlabs.com/products/enzyme-polymer/immpress-hrp-horse-anti-rabbit-igg]
8. Primary CD4 antibody application for FC was quality tested by BioLegend [https://www.biolegend.com/en-us/products/brilliant-violet-605-anti-human-cd4-antibody-7820]
9. PE anti-DYKDDDDK Tag Antibody application for FC was quality tested by BioLegend [https://www.biolegend.com/en-us/products/pe-anti-dykddddk-tag-antibody-9383]
10. Primary CD137 antibody application for FC was quality tested by BioLegend [https://www.biolegend.com/en-us/products/percp-cyanine5-5-anti-human-cd137-4-1bb-antibody-6305]

11. Primary CD25 antibody application for FC was quality tested by BioLegend [https://www.biolegend.com/en-us/products/brilliant-violet-421-anti-human-cd25-antibody-7139]
13. Rat anti-mouse CD16/CD32 application for FC was assessed according to PMID: 8406898
14. Anti-human EpCAM-PECy7 antibody application for FC was quality tested by BioLegend [https://www.biolegend.com/en-us/products/pe-cyanine7-anti-human-cd326-epcam-antibody-8107]
15. Anti-human CD95-BUV395 application for FC was qualified by BD Biosciences [https://www.bdbiosciences.com/en-us/products/reagents/flow-cytometry-reagents/research-reagents/single-color-antibodies-ruo/buv395-mouse-anti-human-cd95.740306]
16. Anti-mouse MHC I-Ak-PE application for FC was quality tested by BioLegend [https://www.biolegend.com/en-us/products/pe-anti-mouse-i-ak-abetak-antibody-3]
17. Anti-mouse CD45-APCCy7 application for FC was quality tested by BioLegend [https://www.biolegend.com/en-us/products/apc-cyanine7-anti-mouse-cd45-antibody-2530]
18. Anti-mouse ICAM1-BV421 application for FC was quality tested by BioLegend [https://www.biolegend.com/en-us/products/brilliant-violet-421-anti-mouse-cd54-antibody-19794]
19. Anti-mouse CD31-BV510 application for FC was qualified by BD Biosciences [https://www.bdbiosciences.com/en-us/products/reagents/flow-cytometry-reagents/research-reagents/single-color-antibodies-ruo/bv510-rat-anti-mouse-cd31.740124]
20. Mouse IgG1k-BUV395 application for FC was qualified by BD Biosciences [https://www.bdbiosciences.com/en-us/products/reagents/flow-cytometry-reagents/research-reagents/single-color-antibodies-ruo/bv510-rat-anti-mouse-cd31.740124]
21. Rat IgG2bk-BV421 application for FC was quality tested by BioLegend [https://www.biolegend.com/en-us/products/brilliant-violet-421-rat-igg2b-kappa-isotype-ctrl-7136]
22. Anti-mouse F4/80-APC application for FC was quality tested by BioLegend [https://www.biolegend.com/en-us/products/apc-anti-mouse-f4-80-antibody-4071]

Alternatively, two antibodies have been optimized and validated through previously published work:
1. Primary Lewis Y antibody optimized according to PMID: 19242371
12. IgG3-APC isotype control optimized according to PMID: 1768791

Eukaryotic cell lines

Policy information about cell lines and Sex and Gender in Research

Cell line source(s)	1. Retroviral packaging cell line PG13-LeY-CAR.<br>2. Human PBMCs isolated from normal donor buffy coats sourced from Australian Red Cross Blood Service.<br>3. DU-145 - ATCC<br>4. PC-3 - ATCC<br>5. 22Rv1 - ATCC<br>6. MDA-MB435 - ATCC
Authentication	Fresh cells have been purchased in the last 12 months. No authentication has been performed.
Mycoplasma contamination	We confirm that all cell lines have routinely tested negative for mycoplasma.
Commonly misidentified lines (See ICLAC register)	No commonly misidentified cell lines were included within the study.

Animals and other research organisms

Policy information about studies involving animals; ARRIVE guidelines recommended for reporting animal research, and Sex and Gender in Research

Laboratory animals	Intact 6-8-week-old male non-obese diabetic severe-combined immune-deficient (NSG) mice (RRID:IMSR_JAX:005557). All mice were bred and housed under controlled temperature (22 °C) and lighting (12:12 h light-dark cycle), and were fed a chow diet ad libitum.
Wild animals	The study did not involve wild animals.
Reporting on sex	Findings apply only to male mice.
Field-collected samples	The study did not involve samples collected from the field.
Ethics oversight	All animal care and procedures for the establishment and maintenance of PDXs were performed in accordance with Monash University animal ethics approvals (MARP/2014/085, MARP/2014/119, MARP/2016/016, 17963, 28911). All animal care and procedures for PDX experimentation were performed in accordance with Monash University animal ethics approval 20374 and Peter MacCallum Cancer Centre animal ethics approval E647.

Note that full information on the approval of the study protocol must also be provided in the manuscript.

Flow Cytometry

Plots

Confirm that:

- [x] The axis labels state the marker and fluorochrome used (e.g. CD4-FITC).
- [x] The axis scales are clearly visible. Include numbers along axes only for bottom left plot of group (a 'group' is an analysis of identical markers).
- [x] All plots are contour plots with outliers or pseudocolor plots.
- [x] A numerical value for number of cells or percentage (with statistics) is provided.

Methodology

Sample preparation	Human PDX tissue is digested with Liberase TM enzymes and stained with pre-titrated antibodies in FACS buffer (1 x PBS with 10% FBS, 5nM EDTA) as per methods.
Instrument	Samples were analyzed on the BD® LSR II Flow Cytometer. Cells were separated using the BD FACSAria™ Fusion Flow Cytometer.
Software	Flow cytometry data was analyzed using FlowJo v10.8 software.
Cell population abundance	>100,000 cells were sorted for each population. A 20uL sample of the sorted cells was placed in 100uL of FACS buffer and re-analysed on the flow-cytometer to determine post-sort purity. Samples with >90% post-sort purity were included for downstream analysis.
Gating strategy	Gating strategy for flow cytometry analysis and FACS sorting. Propidium Iodide was added to samples 30 seconds prior to sample uptake.<br>A. Cells were gated on SSC/FSC to exclude debris;<br>B. Cells were then gated based on exclusion of propidium iodide to gate live cells;<br>C. Single cells were then gated based on FSC-H and FSC-A properties to exclude doublet cells<br>Sorted populations are gated based on isotype control staining or fluorescence minus one controls.

- [x] Tick this box to confirm that a figure exemplifying the gating strategy is provided in the Supplementary Information.
